# Supplementary material for: Neonicotinoid Pesticides Affect Developing Neurons in Experimental Mouse Models and in Human Induced Pluripotent Stem Cell (iPSC)-Derived Neural Cultures and Organoids
Source: Cells. 2024 Jul 31;13(15):1295. doi: 10.3390/cells13151295 (PMC11311455; doi:10.3390/cells13151295)
Supplement: Supplementary file 1 [file cells-13-01295-s001.zip › cells-3115611-Supplementary Material.pdf]

Supplementary Material

Neonicotinoid pesticides affect developing neurons in experimental mouse models and in human induced pluripotent stem cells (iPSC)-derived cells and organoids

Mariani A., Comolli D., Fanelli R., Forloni G. and De Paola M.

1. Supplementary Materials & Methods

1.1 Media composition

CCM

Neurobasal medium, 2% B27 Supplement (Gibco™), 2% horse serum (Life Technologies), 0.5 mM L-glutamine, 25 μM 2-mercaptoethanol 1% penicillin/streptomycin, 25 μM Glutamate (Sigma Aldrich Inch.), 10 ng/ml BDNF (Miltenyi Biotec)

DAM

DMEM/F-12, 1% penicillin/streptomycin (Sigma Aldrich Inch.), 10% heat-inactivated fetal bovine serum (FBS, Euroclone).

NDM

Neurobasal medium, 2% B27 Supplement, 1% GlutaMAX™-I Supplement and CultureOne™ Supplement (Gibco™), 200 uM Ascorbic Acid (Sigma Aldrich), 20 ng/mL human recombinant GDNF and BDNF (Miltenyi Biotech)

ADM

DMEM, 1% N2 Supplement, GlutaMAX™-I Supplement (Gibco™) and FBS (Euroclone)

hCS medium

Neurobasal Plus Medium, 2% B-27 Plus, GlutaMAX™-I Supplement, 20 ng/mL human recombinant GDNF and BDNF

1.2 Supplementary Figure 1 (Fig S1)

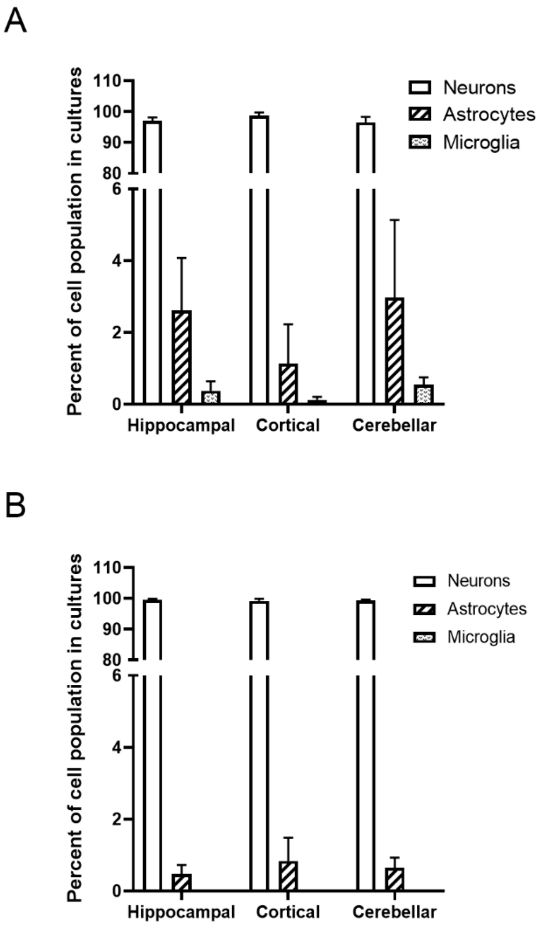

Figure S1. Mouse primary cell culture characterization

Primary neuron cultures were obtained from different areas of mouse embryo brain, and analyzed for the abundance of neurons or glial cells fraction by immunocytochemistry at 6 DIV (**A**) or 14 DIV (**B**) with specific markers for neurons, astrocytes and microglia (NF200, GFAP and IBA-1, respectively).
